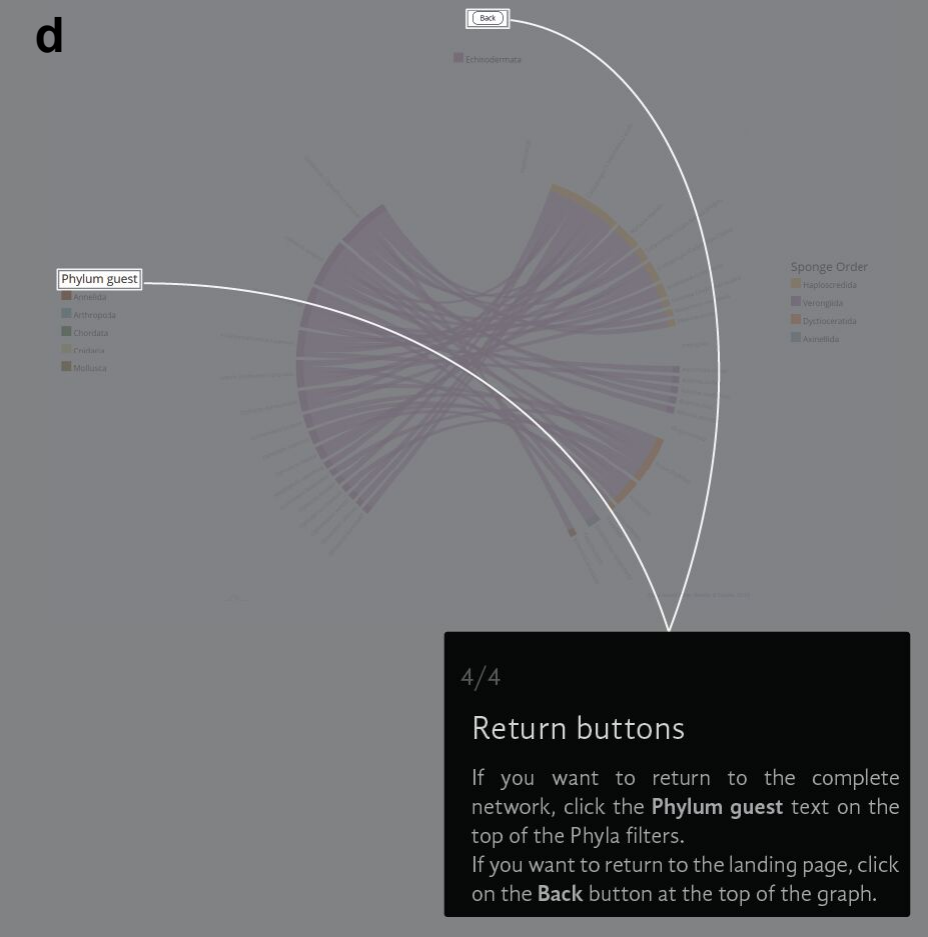# Sponge-dwelling fauna: a review of known species from the Northwest Tropical Atlantic coral reefs

**DOI:** 10.3897/BDJ.9.e63372

**Published:** 2021-03-15

**Authors:** Antar Mijail Pérez-Botello, Nuno Simões

**Affiliations:** 1 Posgrado en Ciencias Biológicas, Facultad de Ciencias, Puerto de Abrigo s/n, C.P. 97356, Sisal, Yucatán, Mexico Posgrado en Ciencias Biológicas, Facultad de Ciencias Puerto de Abrigo s/n, C.P. 97356, Sisal, Yucatán Mexico; 2 Universidad Nacional Autónoma de México, Merida, Mexico Universidad Nacional Autónoma de México Merida Mexico; 3 Laboratorio Nacional de Resiliencia Costera, Mexico, Mexico Laboratorio Nacional de Resiliencia Costera Mexico Mexico; 4 International Chair for Coastal and Marine Studies, Harte Research Institute for Gulf of Mexico Studies, Corpus Christi, United States of America International Chair for Coastal and Marine Studies, Harte Research Institute for Gulf of Mexico Studies Corpus Christi United States of America

**Keywords:** marine ecology, community ecology, interaction networks, symbiosis, mutualism, parasitism, commensalism

## Abstract

**Background:**

Within tropical shallow-water coral reefs, marine sponges provide microhabitats for a wide range of fauna. Although there have been numerous studies and reports of symbiotic relationships amongst sponges and their associated fauna, those pieces of information are isolated and disconnected. For this reason, based on the available literature, we compiled a species-interaction dataset of coral reef marine sponge-associated fauna known to date.

**New information:**

We introduce a dataset that includes 67 literature items that report 101 species of sponge hosts clustered in 12 Orders having a host/guest interaction with 284 guest species from six Phyla present in the Northwestern Tropical Atlantic coral reefs. This dataset consists of two types of information: 1. Machine-readable data and 2. Human-readable data. These two types of coding improve the scope of the dataset and facilitate the link between machine platforms and human-friendly displays. We also created an interactive visualisation of the species-interactions dataset and of a dynamic Chord Diagram of the host-guest species connections to generate a user-friendly link between the user and the dataset.

## Introduction

Symbiosis relationships have been recognised as an important speciation mechanism (Wulff 1997, Watson and Pollack 1999, Hagedorn et al. 2015, Brooker et al. 2019, Bauer 2004). A few years ago, Rossi et al. (2017) introduced the term "Marine Animal Forests" in a book with the same name. In this book, the authors compare the function of trees in forests with marine animal communities like corals, sponges and bivalves. These organisms share one particular characteristic: they can create three-dimensional habitat heterogeneity and structural complexity, providing shelter and a secure food source for a wide range of sessile and mobile animals (Tews et al. 2016, Rossi et al. 2017, Brooker et al. 2019). Sponges tend to be particularly abundant and diverse in coral reef ecosystems. Their architecture, morphology and capability to synthesise toxic substances can generate microhabitats where other species may live or have an adaptive advantage to explore (Bruce and Jones 1976, Duffy 1992, Henkel and Pawlik 2011, Pawlik 2011, Maldonado et al. 2017, Reyes-Bonilla and Jordán-Dahlgren 2017, Koukouras et al. 1995, Maldonado and Young 1996, Diaz and Rützler 2001, Ríos and Duffy 2007).

Reef sponges are a well-studied group; however, the available information on marine sponges' intraspecific relationships is scattered, isolated and, in most cases, is only focused on a particular taxonomic group or a reduced geographical area. For this reason and based on published records from the Northwest Tropical Atlantic (NWTA) coral reefs, we compiled and created a standardised dataset that brings together information on sponge host/guest interactions in the reagion. Moreover, we also created two dynamic and interactive web visualisation tools to describe and analyse the information.

## General description

### Purpose

In a climate change and biological diversity loss scenario, it becomes crucial to have a high-quality open-access baseline dataset on fundamental aspects, such as symbiotic interactions. This dataset provides an updated and standardised matrix of published records on host/guest interaction between tropical coral reef marine sponges and their associated fauna. Each interaction was codified into a machine- and human-readable format, according to the Global Biotic Interactions (GloBI) standard language (for more information, see Poelen et al. 2014; globalbioticinteractions.org). Furthermore, an independent, dynamic, interactive and user-friendly data-visualisation display of this information is provided to maximise outputs in terms of data accessibility and usage.

### Additional information

In this work, we screened 65 articles and two university theses on the NWTA coral reefs published between 1909 and 2019. The present review includes 101 sponge species divided into 12 Orders from the Demospongiae Class, interacting with 284 guest species from six Phyla. The Haplosclerida and Dyctioceratida orders presented the largest number of associated species. Regarding the host sponges morphologies, the tube, fan and vasiform shapes common to the genera *Agelas*, *Aplisyna*, *Ircinia* and *Callyspongia*, tended to have more guest species. *Ircinia strobilina* was the species with the highest number of associated species (dwelling species N = 89) followed by *Callyspongia aculeata* (dwelling species N = 63) and *Ircinia felix* (dwelling species N = 53). According to the sponges-dwelling fauna records, the phylum Arthropoda was dominant on species numbers (164 spp.), followed by Annelida (60 spp.), Mollusca (19 spp.), Chordata (20 spp.), Echinodermata (15 spp.) and Cnidaria (6 spp.).

When we compare the host/guest species richness between the Caribbean and the Gulf of Mexico, the Caribbean has the greatest sponge diversity with 84 sponge species, whereas, the Gulf of Mexico has 38 sponge species. Both regions share 22 sponge species. However, the Gulf of Mexico has 191 guest species against 145 guest Caribbean species. At the guest species richness part, both regions shared 52 guest species. If we count the interaction diversity (an integrated binomial of host/guest species), the current work register 451 host/guest interaction within the Gulf of Mexico, but only 399 inside the Caribbean coral reefs.

Meanwhile, mutualistic associations are a common interaction type with 86 entries, followed by the parasitic interaction with 44 entries and commensal interaction with 36 entries. Nonetheless, most of the literature entries do not classify the type of interaction, remaining at the symbiosis or dwelling-species level.

Sponges, like other bio-constructing species, are ecosystem engineers, shaping the environmental complexity and maintaining part of the habitat biodiversity (Jones et al. 1994, Rossi et al. 2017). The present species-interactions' dataset highlights the remarkable diversity of animals that depend on, or take advantage of, the sponges' presence. Besides, it is possible to have a host/guest distribution, host/guest species richness quantification and a few more quantitative metrics that will help to better understand and model the sponge-dwelling fauna.

## Sampling methods

### Sampling description

In order to perform the literature search and compilation of the interaction dataset, based on bibliographic records, it was necessary to define our sampling unit. For this work, we define each article and thesis reviewed as a unit; each of these elements we name as "literature item" and each item could provide one or several interaction report entries.

First, we compiled all the articles and theses known to us that report a sponge host/guest interaction in the NWTA coral reefs (known literature items). This initial baseline was complemented with a Web of Science, Pub Med, Crossref, Scopus and Google Scholar web search (web literature items) using the "Publish or Perish" software application (Harzing 2007; harzing.com/resources/publish-or-perish). A specific string of keyword sequences and logic operators was used to simultaneously focus the search without losing inclusiveness and to improve the exploration yield [(“Sponge”) AND (“dwelling” OR “interaction” OR “association” OR “mutualism” OR “commensalism” OR “parasitism”) AND (“Annelida” OR "Arthropoda" OR "Chordata" OR "Cnidaria" OR "Echinodermata" OR "Mollusca" OR "Molluska") AND (“coral reef”) AND (”Caribbean” OR “Gulf of Mexico” OR “Northwestern Atlantic”)].

To identify possible duplicate and pseudoreplicate literature items between the web search and the known literature items, we used the "Check for Duplicates" tool implemented in Mendeley software (mendeley.com ). This tool compares the publication type (Journal Article or Thesis), the literature title, authors, publisher and publication year for all the literature items within the bibliographic database. With this comparison, it was possible to discriminate both duplicate and pseudoreplicates literature items.

A literature item would be validated 1. if it were published in an indexed journal, according to the Science Citation Index Expanded (SCIE) or in a MSc or PhD University theses; 2. if the literature item were an indexed journal, necessarily had to match the geography of interest, contain details of the latitude and longitude information (or a detailed geographical description) and clearly stated the species involved. If the literature item were a University thesis, the previous criteria were used, but it was also indispensable that the species involved were deposited in a scientific collection. With this protocol, we ensured that all complied literature items has the minimum essential information to be extracted. Whenever possible, the interaction type presence (commensalism, parasitism, mutualism), the species taxonomy details and the host body part where the guest lived, were also extracted. Finally, with the screened literature items, we compiled the sponge-dwelling fauna dataset. The compiling process consisted of generating independent entries, based on the sponge host/guest interaction reports inside a particular item.

### Quality control

Data were standardised according to the GloBI standard language. This guideline consists of categorising each entry into different standardised vocabularies. We cross-checked the species scientific names with the World Register of Marine Species webserver (Costello et al. 2013; WoRMS; marinespecies.org/aphia.php?p=match), retrieving the actual classification and the universal identifier, Aphia ID, provided by the UN-Global Biodiversity Information Facility. The geographic information was integrated and codified according to the GeoNames ID platform (geonames.org). The interaction type and host body part name were standardised, according to the OBO Library (obofoundry.org). Lastly, for the reference management and citation style, we used Mendeley software. With this standardisation and quality control process, we ensured a high-quality integrated human-readable and machine-readable dataset.

### Step description

Step 1: Define the sampling **universe**; this step was designed to mark the geographic and environmental limits.

Step 2: Literature **search**; in this step, we compiled the curated bibliographic database, without duplicates and pseudoreplicates between the known literature items and the web search literature items.

Step 3: Item **validation**; this step consists of a validation test that we used to select the literature items with the minimum necessary information.

Step 4: Entry **standardisation**; in this step, we homogenised all the sponge iteration entries into the GloBI standard language.

**Figure 1.**
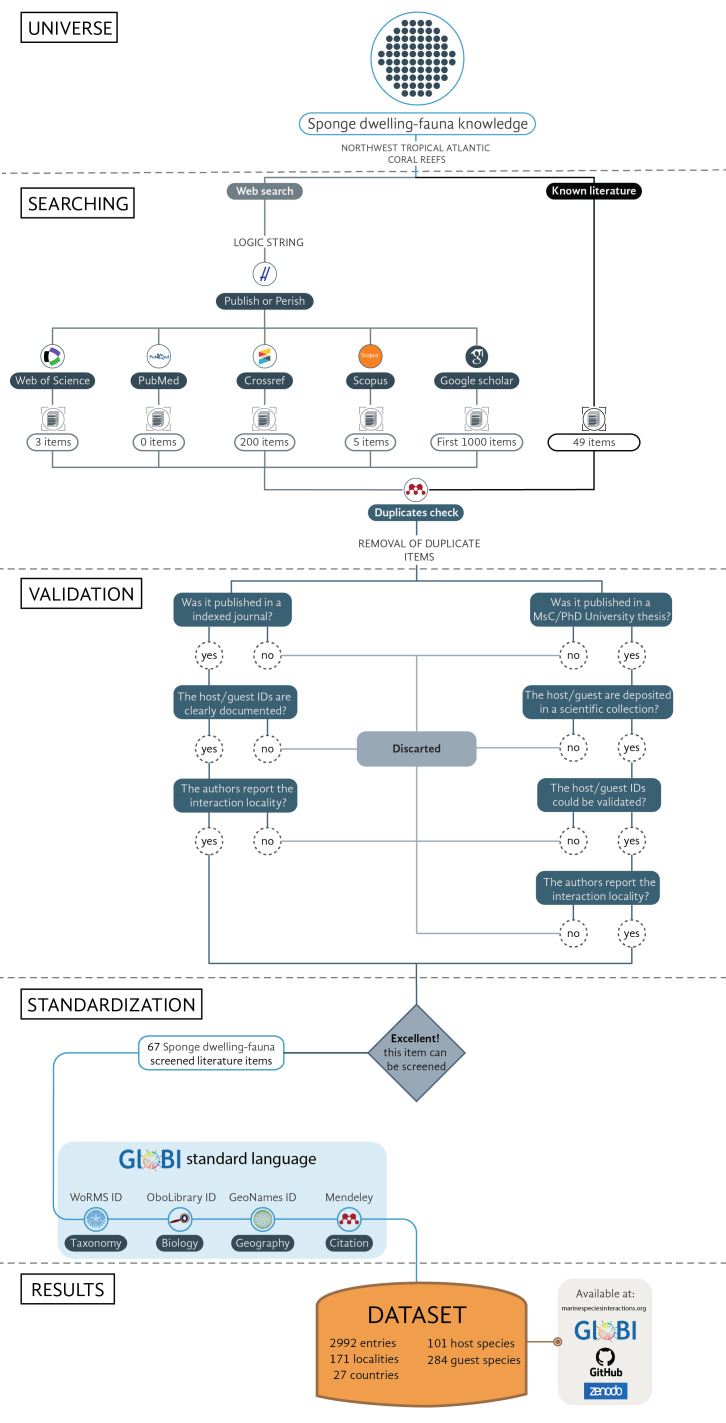
Flow diagram of the steps followed to generate this dataset.

Step 5: Dataset compilation (Fig. 1)

## Geographic coverage

### Description

According to the large marine ecosystems' classification proposed by Spalding et al. (2007), the Northwestern Atlantic has five regions with major coral reef formations: the Gulf of Mexico, the Caribbean Sea formed by the greater and Lesser Antilles, Central America and the north shores of South America, North America, the Bahamian Archipelago and Bermuda at the north-eastern boundary of this major region.

## Taxonomic coverage

### Description

This dataset is composed of the host/guest interaction between coral reef sponges (Pylum: Porifera) and six other major marine Phyla: Arthropoda, Annelida, Mollusca, Chordata, Echinodermata and Cnidaria. All the information is at species resolution.

### Taxa included

**Table:** 

Rank	Scientific Name	Common Name
phylum	Porifera	Sponges
phylum	Arthropoda	Shrimps, crabs, lobsters
phylum	Annelida	Worms, christmas tree worm
phylum	Mollusca	Clams, mussels, oysters and scallops
phylum	Chordata	Fish, goby
phylum	Echinodermata	Sea urchins, sea cucumbers, brittle-stars
phylum	Cnidaria	Sea anemones, hydroids

## Temporal coverage

**Data range:** 1909-1-01 – 2019-12-31.

## Usage licence

### Usage licence

Creative Commons Public Domain Waiver (CC-Zero)

## Data resources

### Data package title

Sponge dwelling-fauna from the North-western Tropical Atlantic Ocean: a bibliographic records database.

### Resource link

https://doi.org/10.5281/zenodo.3333023

### Alternative identifiers

https://github.com/BDMYRepository/Sponge_Interactions

### Number of data sets

1

### Data set 1.

#### Data set name

Sponge-dwelling fauna from the North-western Tropical Atlantic Ocean: a bibliographic records database.

#### Data format

.tsv

#### Number of columns

30

#### Download URL

https://zenodo.org/record/3333023

#### Data format version

2.06

#### Description

**Figure 2.**
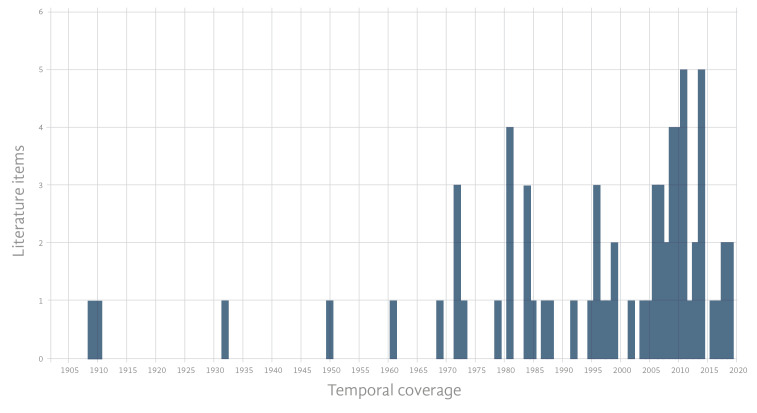
Distribution of the literature items within the dataset temporal coverage.

The present database compile 65 articles (Baeza et al. 2016, Carrera-Parra and Vargas-Hernández 1997, Chace 1972, Chavarro et al. 2004, Böhlke and Robinson 1969, Cházaro-Olvera and Vázquez-López 2014, Christoffersen 1972, Coutière 1909, Coutière 1910, Crocker and Reiswig 1981, Crowe and Thomas 2002, D'Aloia et al. 2011, Dardeau 1981, Dardeau 1984, Dauer 1973, Duffy 1992, Duffy 1996a, Duffy 1996b, Duffy 1996c, Duffy 1998, Duffy and Macdonald 1999, Erdman and Blake 1987, García-Hernández and Hoeksema 2017, Hendler 1984, Henkel and Pawlik 2005, Henkel and Pawlik 2011, Henkel and Pawlik 2014, Herrick 1981, Hultgren and Duffy 2010, Huang et al. 2008, Hultgren et al. 2011, Hultgren et al. 2010, Lattig and Martin 2009, Lattig and Martín 2011, LeCroy 1995, Macdonald and Duffy 2006, Macdonald et al. 2009, Macdonald et al. 2006, Montenegro-González and Acosta 2010, Ortiz et al. 2011, Ortiz et al. 2013, Paerse 1932, Pearse 1950, Pawlik 2011, Pequegnat and Heard 1979, Randall and Lobel 2009, Rebolledo et al. 2014, Reimer et al. 2018, Richards et al. 2007, Rios and Duffy 1999, Robertson and Tassell 2019, Santana-Moreno et al. 2013, Scott et al. 1988, Swain 2012, Swain and Wulff 2007, Thomas and Klebaa 2007, Thomas and Klebba 2006, Tobb and Manning 1961, Töth and Bauer 2008, Tyler and Böhlke 1972, Victor and Krasovec 2018, Villamizar and Laughlin 2011, Westinga and Hoetjes 1981, Williams 1984, Winfield et al. 2009, Winfield and Ortiz 2010, Wendt et al. 1985) and two university theses (Ugalde García 2014, Perez-Botello 2019) in a detailed sponge host-guest interaction dataset distributed in the Northwest Tropical Atlantic coral reefs, including a total of 2992 interactions between 101 sponge host species and 284 sponge-dwelling species, over 90 years of publications (Fig. 2). All entries are standardised to the GloBI language.

**Data set 1.** 

Column label	Column description
sourceOccurrenceId	Globally unique id to reference the individual source organism.
sourceTaxonId	Taxon classification id of originating organism in some taxon name authority. WoRMS AphiaID
sourceTaxonName	Scientific name of taxon classification of source organism
sourceBodyPartId	Identifier of description of source body part is interacted with. As described by the OBO Relations Ontology
sourceBodyPartName	Human-readable description of source body part
sourceLifeStageId	Identifier of description of source life stage. As described by the OBO Relations Ontology
sourceLifeStageName	Human-readable description of source life stage
interactionTypeId	Id of interaction. As described by the OBO Relations Ontology
interactionTypeName	Human-readable description of interactions
targetOccurrenceId	Globally unique id to reference the individual target organism
targetTaxonId	Taxon classification id of target organism. WoRMS AphiaID
targetTaxonName	Scientific name of taxon classification of target organism of interaction
targetBodyPartId	Identifier of description of target body part is interacted with. As described by the OBO Relations Ontology
targetBodyPartName	Human-readable description of target body part.
targetLifeStageId	Identifier of description of target life stage. As described by the OBO Relations Ontology
targetLifeStageName	Human-readable description of target life stage.
localityId	Identifier of the Geo classification. As described by geonames.org
localityName	Human-readable description of locale
decimalLatitude	Latitude of geographic centre of interaction observation location
decimalLongitude	Longitude of geographic centre of interaction observation location
YYYY	Year of the recorded interaction
MM	Month of the recorded interaction
DD	Day of the recorded interaction
HH	Hour of the recorded interaction
mm	Minute of the recorded interaction
ss	Second of the recorded interaction
observationDateTime	ISO 8601 formatted date time string of the recorded interaction
referenceDoi	Digital Object Id used to the papers, datasets or other digital object that validate the interaction
referenceUrl	Some resolvable url that points to information related to species interaction record
referenceCitation	Human-readable reference related to species interaction record

## Additional information

### Interactive display and data visualisation

A virtual environment was generated to visual-analyse the dataset. We created a Tableau dashboard (public.tableau.com) and a AmCharts Chord Diagram (amcharts.com/demos/toggleable-chord-diagram). Both the interactive dashboard and the dynamic Chord Diagram are available at the project official web page: marinespeciesinteractions.org/projects/visual-database/. The uses of the interactive dashboard are based on different lists that filter the displayed information according to the users' requests. The dashboard shows a map of the NWTA where the records of each interaction are plotted (Fig. 3a). In the middle are 10 filters with host Order, Family and Scientific species name, guest Phyla, Class, Order, Family and Scientific species name, the recorded locality (country) and the information source (Fig. 3b). On the right side, two bar graphics show either the sponge Order vs. guest species richness or the guest Phylum vs. guest Class species richness counts (Fig. 3c, d). The host/guest matrix is centred in the lower part of the dashboard, with the host sponges as rows and the sponge dwelling-fauna species as columns (Fig. 3e). In practice, the interactive dataset aims to be an intuitive step-by-step graphical interface. It is possible to select the source of information to observe and focus on a particular region or taxonomic group.

The Chord Diagram gives a general picture of everything in the universe of registered interactions (Fig. 4a). The thickness of the node represents the number of links that a species has and the colour represents the taxonomic group to which it belongs. The information can be filtered by guest Phylum (i.e. Annelida, Arthropoda, Chordata, Cnidaria, Echinodermata and Mollusca) (Fig. 4b), but not by the sponge Order (Fig. 4c). If the user wants to return to the original view, they can click on the guest Phylum name or the back button (Fig. 4d).

Although the complete dataset is fully accessible for downloading as a whole, with these two interactive visualisation tools, openly available through the internet and hopefully sufficiently intuitive, the user can interact with the dataset and pose questions filtered according to their particular interest.

### What’s next?

With this dataset, we provide an updated and clustered report on the symbiotic relationships in coral reef sponges in the NWTA coral reefs. This information opens the door to many numerical and statistical analyses. Finally, we encourage you to collaborate with this project and, if you have any records that are not listed on this dataset, contact us. We will be glad to talk with you and add this information in the next version of the Dataset.

### Concluding remarks

Compiling the available sponge host/guest interaction data in one place enhances the scope and shareability of the diffused information. Furthermore, the standardisation of the dataset into a global language creates a link between this dataset and several international repositories, such as The Encyclopaedia of Life and communication with other data languages, such as Darwin Core. Moreover, with this work, a baseline is generated to compare and structure future works that focused on sponge host/guest relationships.

In conclusion, the state of knowledge about sponge-associated fauna is on the right path. However, the main obstacle during the data collection process was the lack of reported information. For example, the involved species' taxonomic identity and the interaction type are crucial pieces of information that are missing in several literature items. We suggest that future works make an effort to clearly identify both taxonomic entities, not only the guest or host species. Furthermore, it was possible to analyse the interaction matrix of sponge-dwelling species with a complex network approach identifying connected and key species with this dataset. To better understand possible changes in the sponge host/guest interactions, a niche-modelling approach could also be useful, displaying different future species-interaction scenarios.

## Figures and Tables

**Figure 3a.**
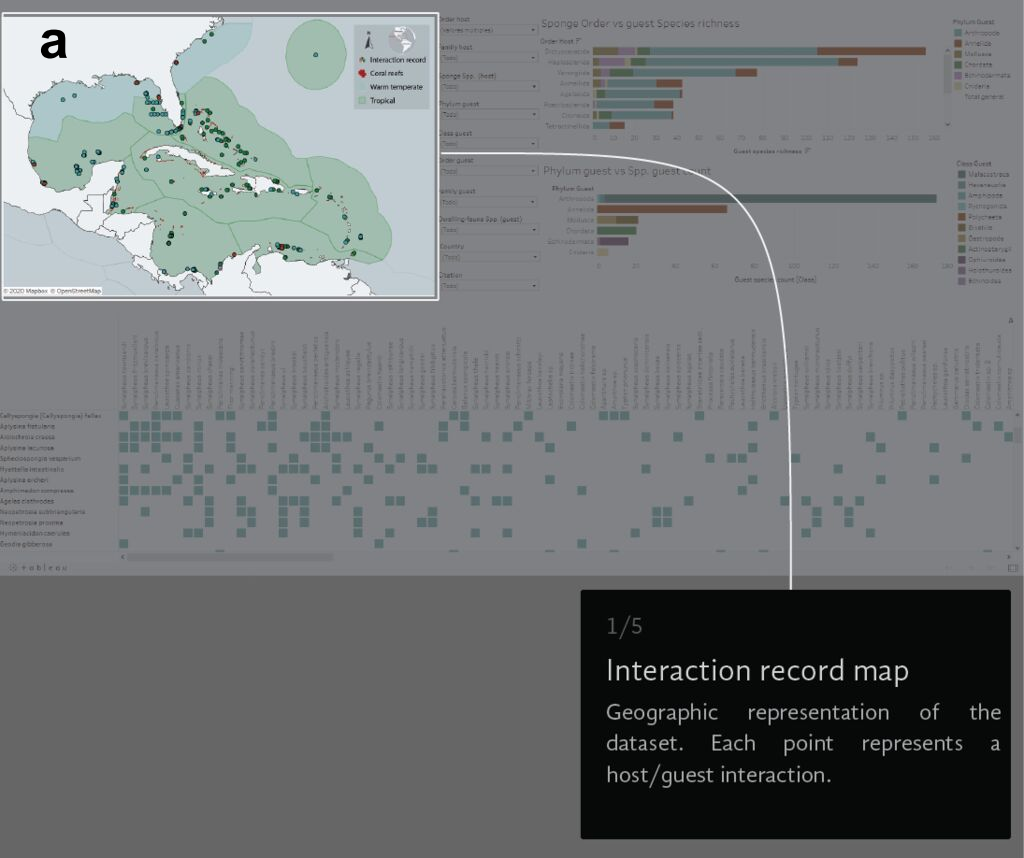


**Figure 3b.**
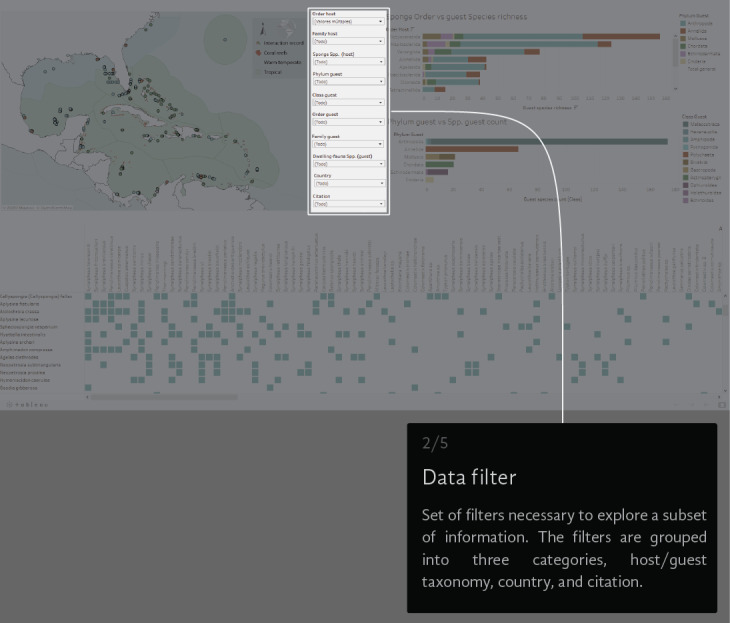


**Figure 3c.**
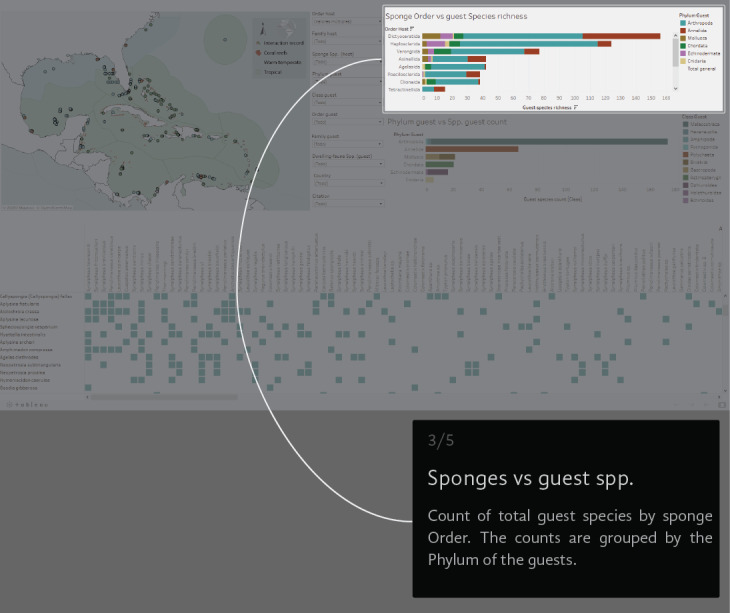


**Figure 3d.**
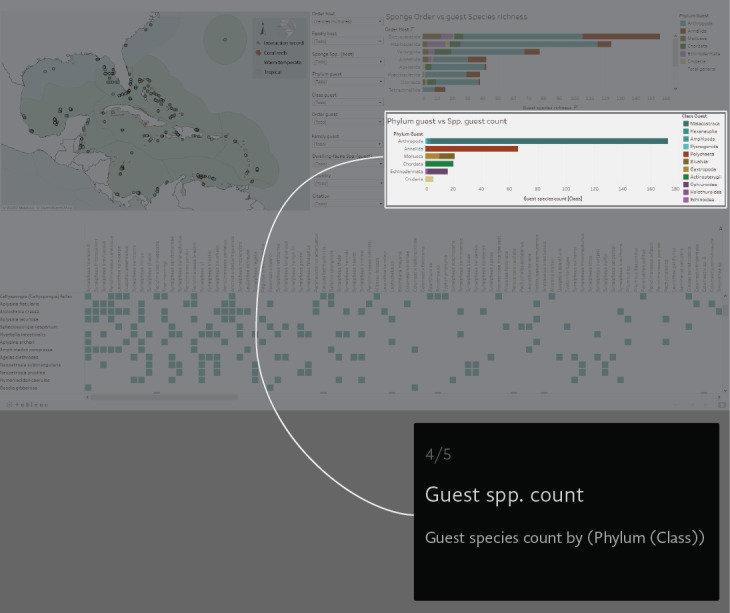


**Figure 3e.**
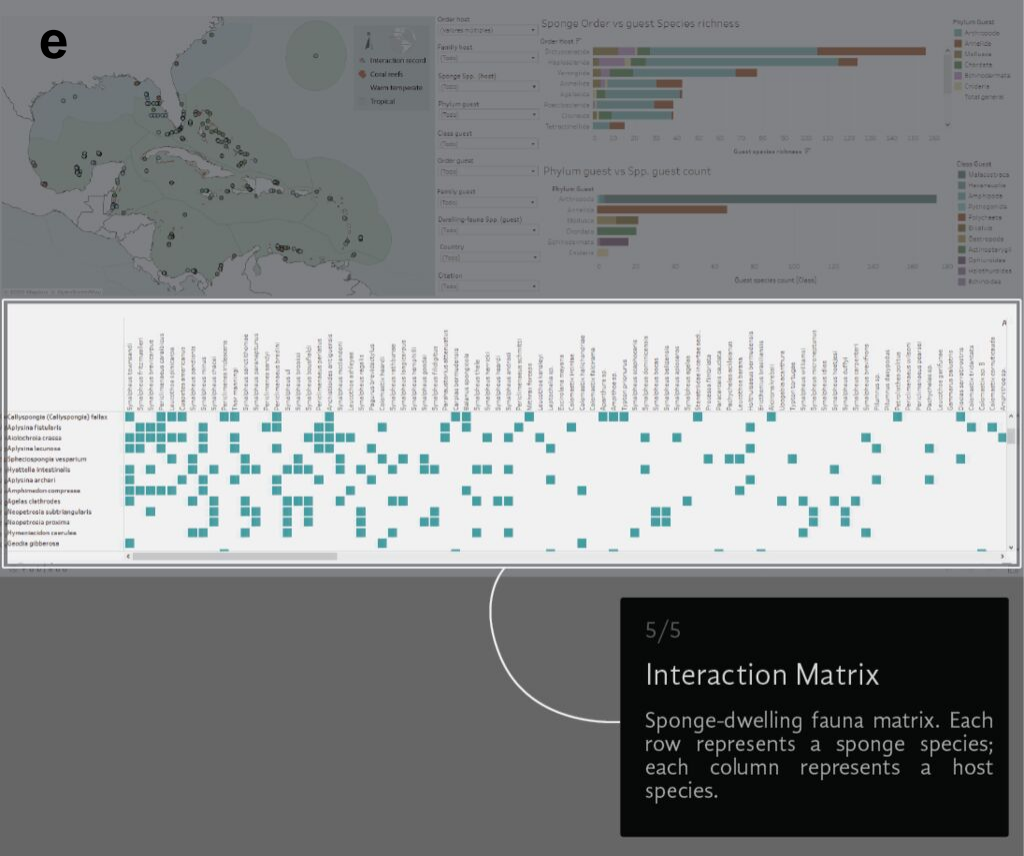


**Figure 4a.**
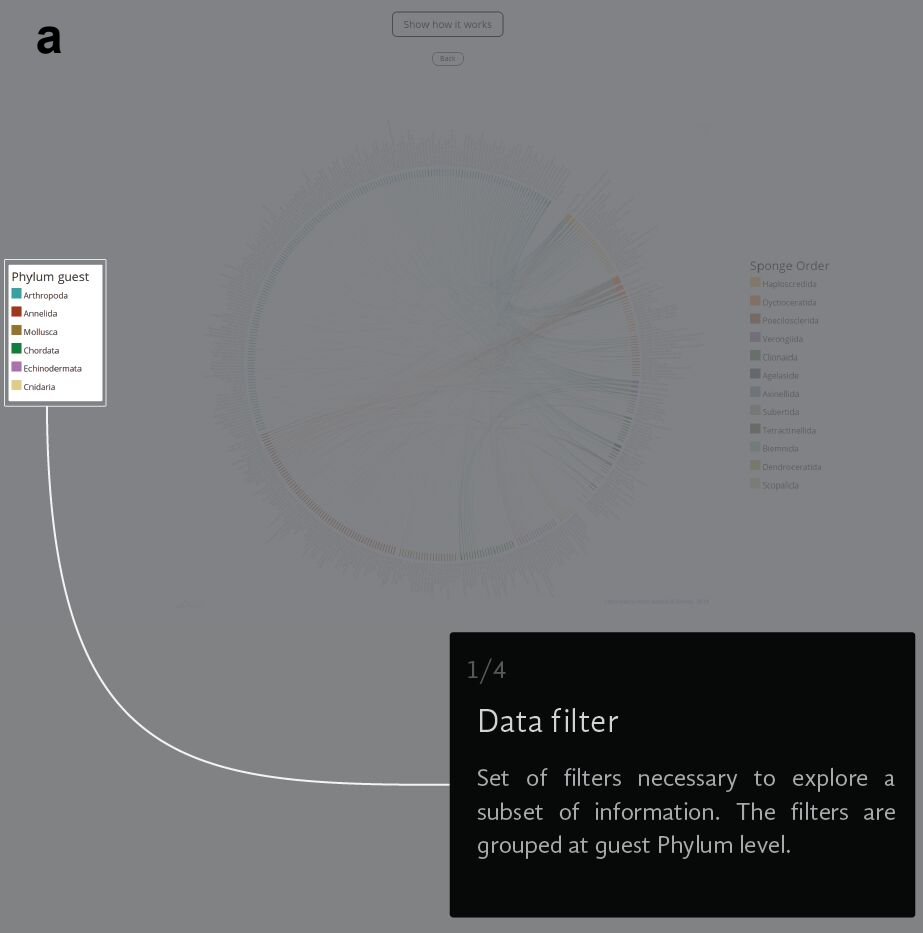


**Figure 4b.**
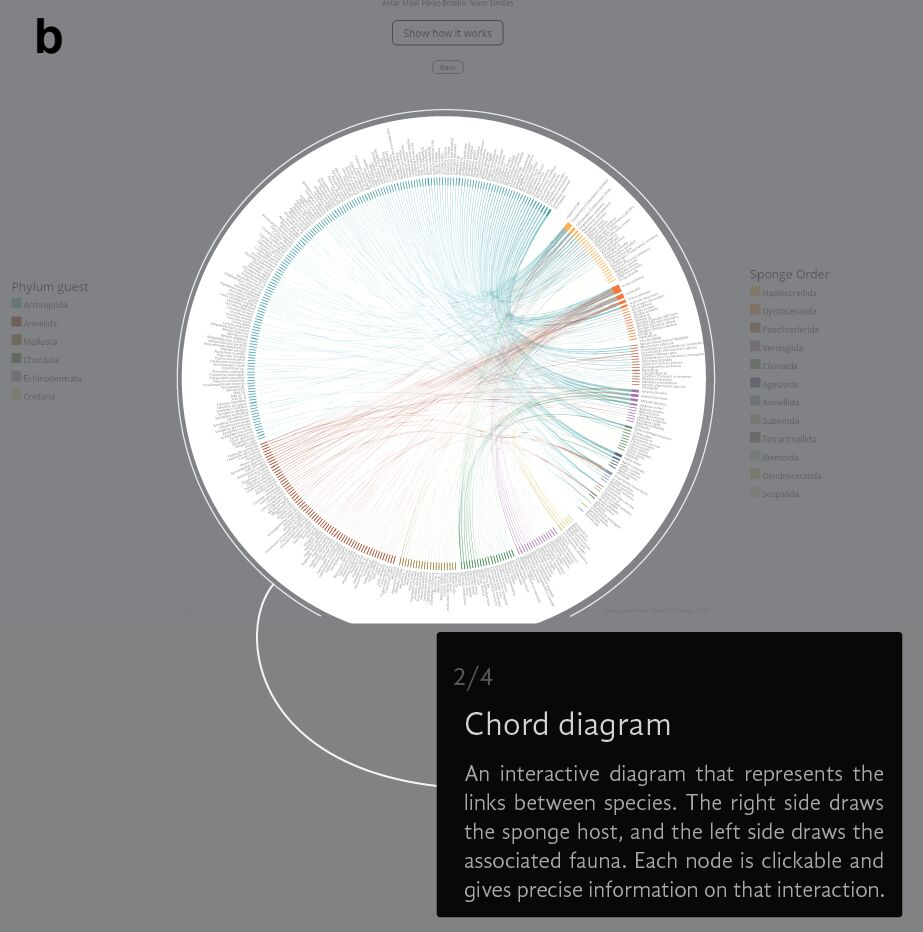


**Figure 4c.**
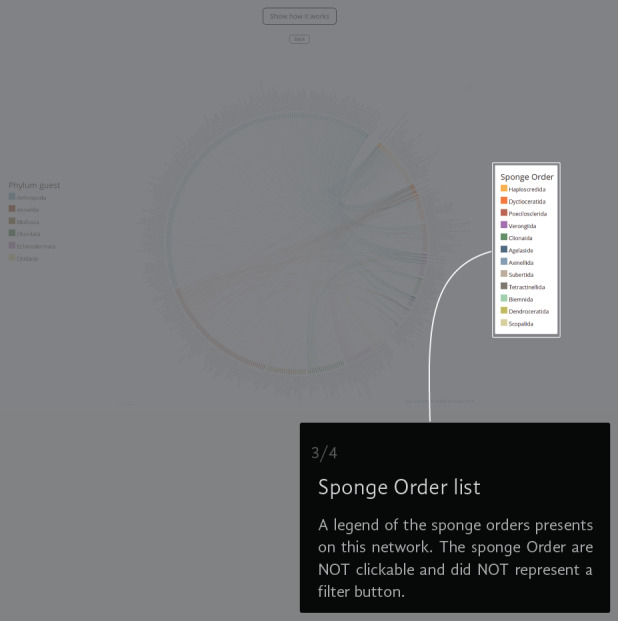


**Figure 4d.**